# Microstructure and Tensile Property of Laser Cladding Assisted with Multidimensional High-Frequency Vibration

**DOI:** 10.3390/ma15124295

**Published:** 2022-06-17

**Authors:** Zhehe Yao, Jian Chen, Hongyu Qian, Yanbin Nie, Qunli Zhang, Jianhua Yao

**Affiliations:** 1 College of Mechanical Engineering, Zhejiang University of Technology, Hangzhou 310023, China; zhyao@zjut.edu.cn (Z.Y.); jianchen@zjut.edu.cn (J.C.); hongyuqian@zjut.edu.cn (H.Q.); 2111902260@zjut.edu.cn (Y.N.); zql@zjut.edu.cn (Q.Z.); 2 Institute of Laser Advanced Manufacturing, Zhejiang University of Technology, Hangzhou 310023, China; 3 Collaborative Innovation Center of High-End Laser Manufacturing Equipment (National 2011 Plan), Hangzhou 310023, China

**Keywords:** laser cladding, multidimensional high-frequency vibration, microstructure, microhardness, tensile strength

## Abstract

Laser cladding is a promising surface modification technology to fabricate high-performance parts. However, defects such as porosity, cracks and residual tensile stress are easily produced in laser cladding, leading to significant property reduction and poor reliability. In this study, laser cladding with multidimensional high-frequency vibration was investigated. The effects of multidimensional high-frequency vibration on the improvement of microstructure and mechanical properties were analyzed and discussed based on the vibration-assisted laser cladding experiments. In addition, a numerical model was conducted to help understand the significance of the vibration on flow field and temperature field. Results show that 3D vibration led to the primary dendrite spacing reduction from 11.1 to 6.8 μm, microhardness increase from 199 to 221 HV_0.2_, and a nearly 110% improvement in the elongations. The findings of this study confirmed the significant benefits of multidimensional high-frequency vibration applied in laser cladding and provided a basis to uncover the underlying mechanisms of multidimensional vibration on the rapid melting and solidification.

## 1. Introduction

Laser cladding, as a promising surface modification technology, has been widely applied in the fields of aerospace [1,2], manufacturing [3,4], medical [5], etc., due to the advantages of high power, acceptable heat-affected-zone and small distortion of the substrate [6,7]. On the other hand, it is still difficult to obtain high-quality laser cladding due to the microstructure characteristics and the defects generated in the laser cladding processes [8]. Therefore, numerous research studies have been carried out to improve the microstructures and properties. Manjaiah et al. [9] investigated the effects of processing parameters including laser power and scanning speed on the morphology and quality of the coatings, and they found that the microhardness of samples fabricated by optimized parameters was similar to that of as-cast and wrought samples. Shan et al. [10] adopted the “material genetic” design method to optimize the Fe-based alloy composition, resulting in a laser cladding layer with high wear and corrosion resistance. Sun et al. [11] adopted an industry-used broad-beam laser to prepare the crack-free coating based on process studies, and the coating had great anti-friction property and excellent wear resistance compared to the substrate.

The studies above focus on improving the cladding properties by adjusting the processes of the cladding. Various types of external energy, including electromagnetic fields [12], induction heating [13], vibration [14,15,16,17], etc., have been applied to assist the laser cladding processes. Among them, vibration is able to provide various effects, including grain refinement, pore suppression, microstructure homogenization, etc., during the solidification process [18]. Several studies have adopted vibration in assisting the laser cladding as follows. Foroozmehr et al. [14] found a coating with an 80% reduction in porosity fabricated by vibration-assisted laser cladding. Liu et al. [15] investigated the effects of vibration frequency on the coating mechanical property and found that hardness and corrosion were both improved firstly and reduced subsequently with the increase in vibration frequency. Li et al. [16,17] suggested that the microstructure of a coating can be effectively refined by applying high-frequency vibration. Compared to the single dimensional vibration used in the above studies, multidimensional high-frequency vibration is able to apply significant stir in the molten pool other than oscillation, which will improve the comprehensive performance of the coating [19]. To the best of the authors’ knowledge, there are still few reports on multidimensional vibration-assisted laser cladding.

In this study, multidimensional high-frequency vibration was adopted in the laser cladding of stainless steel 316L (SS316L) to investigate the microstructure and tensile property. A numerical model of vibration-assisted laser cladding was established to understand the flow field and temperature field in the molten pool. Comparative studies on the microstructures and the mechanical properties of the cladding layer with and without vibration were conducted to reveal the effects of multidimensional vibration on laser cladding.

## 2. Materials and Methods

### 2.1. Materials

**Figure 1:**
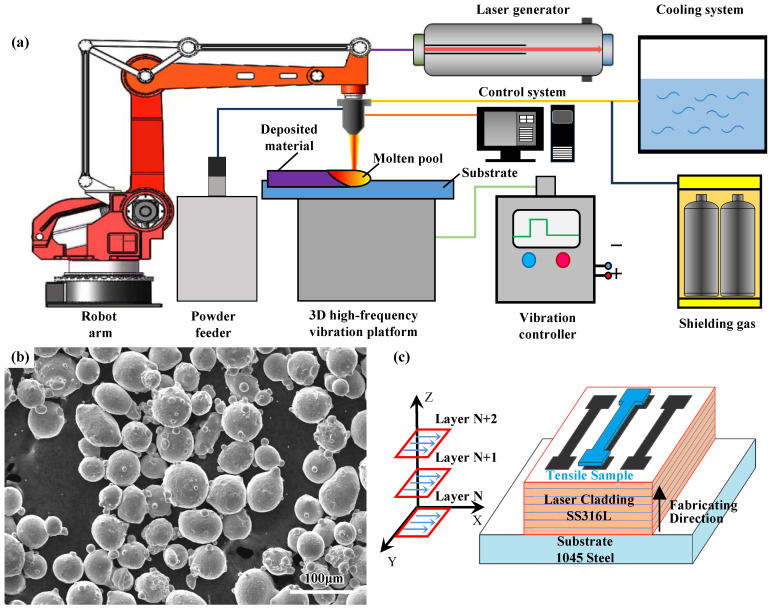
The experimental setup and materials: (**a**) diagram of the experimental setup; (**b**) SEM micrograph of SS316L powders; (**c**) schematic illustration of the sampling for tensile tests.

SS316L powders were adopted for the cladding layer due to its moderate price, strong corrosion resistance and great self-melting property. The SS316L spherical powders (Zhejiang Asia General Soldering & Brazing Material Co., Ltd., Zhejiang, China) with a particle size range between 48 and 75 µm were used in this study. Before experiments, the powders were baked in an oven at 120 °C for 30 min. The powder morphology is shown in Figure 1b. For the substrates, 1045 steel plates (Shanghai Shanxiong Special Steel Co., Ltd., Shanghai, China) with dimensions of 100 mm × 10 mm × 20 mm were used. Prior to the cladding process, the substrates were polished by abrasive paper to remove the oxide layer and then cleaned by alcohol. The chemical compositions of the 1045 steel plates and the SS316L powders are shown in Table 1, which were from the data sheets provided by the suppliers.

### 2.2. Experimental Setup and Procedures

The experimental setup mainly consisted of a LDF6000-40 high power flexible fiber-coupled diode laser manufacturing system, a three-degree-of-freedom high-frequency vibration platform, and a motion device using an IRB2400/16 robot. The output wavelength of the laser is 940~1060 nm and the highest output power is 6000 W. The substrate was fixed on the vibration platform. The vibration was generated from the vibration platform and transmitted from the bottom of the substrate to the molten pool. The schematic of multidimensional vibration-assisted laser cladding system is shown in Figure 1a.

In this study, laser cladding experiments were carried out with the superimposition of various vibration directions including one-dimensional vibration (1D vibration, vertical), two-dimensional vibration (2D vibration, vertical + parallel) and three-dimensional vibration (3D vibration, vertical + parallel + lateral). The cladding parameters were optimized by an orthogonal experimental design prior to this study. The cladding with no obvious defects, appropriate values of the dilution rate and the width-to-height ratio were considered as the optimization goals. Then, a laser power of 1600 W, a spot diameter of 4 mm, a scanning velocity of 5 mm/s, and a powder feeding rate of 6 g/min were selected to investigate the effects of vibration on laser cladding.

A high-frequency vibration meter (Xima AR63B) was used to test the acceleration response of the substrate with different frequencies, and a resonant frequency of 2196 Hz was selected in this study. The working power of the vibration platform is 2.2 kW. The vibration waveform is sine wave, and the acceleration amplitude of the vibration is 29.6 m/s^2^. Argon gas with the purity of 99.99% was used for delivering powder and shielding the processing.

### 2.3. Testing Approach

The metallographic samples were cut from the cladding layer through the cross-section of the specimens using a wire electrical discharge machining. Then, the samples were polished and etched according to standard procedures. The microstructures were observed using an optical microscope (Axio Scope. A1, ZEISS) and scanning electron microscopy (SEM, ZEISS EVO 18). The microhardness of the claddings was measured using a digital micro-hardness tester (HMV-2TADWXY) with a load of 1.961 N and 10 s holding time along the cladding thickness (depth). Each experiment was repeated at least three times.

Multi-layer cladding was prepared for the tensile tests. The overlapped cladding layer samples were fabricated, and the overlapping coefficient used was 50% with the sequential scan mode. The direction of the single-pass scanning speed (X direction) was perpendicular to the direction of the cladding overlap (Y direction). The sampling position for tensile tests was along the transverse overlapping direction, as shown in Figure 1c. The tensile tests were tested by an electronic universal testing machine (CMT5105-100kN) at room temperature with a loading speed of 0.5 mm/min. The fracture surfaces of the broken tensile samples were observed by SEM.

## 3. Numerical Studies

The heat transfer and fluid flow in the molten pool significantly affect the solidification microstructure and the mechanical properties [20]. Since laser cladding is a transient process, it is quite difficult to wholly measure the evolution of the temperature and the flow field in the molten pool by sensors. Therefore, numerical studies were adopted to understand the dynamic behavior of heat transfer and fluid flow in the molten pool.

### 3.1. Assumptions

Considering the complexity of vibration-assisted laser cladding (melt, additive, fluid flow and solidification), several assumptions were used [21,22,23] and listed as follows.

(1) The fluid flow in the molten pool is assumed to be Newtonian, incompressible and laminar.
(2) The fluid inside the molten pool is subjected to buoyancy, which is in accordance with the Boussinesq hypothesis.
(3) The mushy zone where the temperature is between the solidus and liquidus is assumed as a porous medium with isotropic permeability.
(4) The powder flow is assumed to have a Gaussian distribution, and the powder is melted when it falls in the molten pool.

### 3.2. Mathematical Model

The equations of mass conservation, momentum conservation, and energy conservation used in the model are described as follows [24].

Mass conservation equation:

(1) $$\frac{\partial \rho }{\partial t}+\nabla \cdot \left(\rho u\right)=0,$$

where *ρ* is the density, *t* is the time, and **u** is the velocity vector of fluid.

Momentum conservation equation:

(2) $$\rho \frac{\partial u}{\partial t}+\rho \left(u\cdot \nabla \right)u=\nabla \cdot \left[-pI+\mu \left(\nabla u+{\left(\nabla u\right)}^{T}\right)\right]+F_{\mathrm{Bouyancy}}+F_{\mathrm{Darcy}},$$

where *μ* is the melt viscosity; **F_Bouyancy_** denotes the buoyancy force caused by density gradient because of temperature gradient. **F_Bouyancy_** is usually described using the Boussinesq approximation [24] as follows.

(3) $$F_{\mathrm{Bouyancy}}=\rho g\beta \left(T-T_{\mathrm{ref}}\right),$$

where *β* is the coefficient of thermal expansion, *T*_ref_ is the reference temperature of the metal, and *T*_ref_ is the melting point.

The Darcy term **F_Darcy_** represents damping force, which is the approximation of the Carman–Kozeny equation derived from Darcy’s law to describe the momentum dissipation of the mushy zone. It can be written in the following form:

(4) $$F_{\mathrm{Darcy}}=-A_{\mathrm{mush}}\frac{\left(1-f_{l}^{2}\right)}{f_{l}^{3}+B}\times u,$$

where *A*_mush_ is a constant that depends on the morphology of the porous medium; *B* represents a small number to avoid division by zero. *f*_l_ denotes the liquid mass fraction, which is defined as:

(5) $$f_{l}=\left\{ \begin{array}{ll} 1 & \;\;T>T_{l}\\ \frac{T-T_{s}}{T_{l}-T_{s}} & \;\;T_{s}\leq T\leq T_{l}\\ 0 & \;\;T<T_{s} \end{array}\right.,$$

where *T*_s_ is the solidus temperature, and *T*_l_ is the liquidus temperature.

Energy conservation equation:

(6) $$\rho C_{p}u\cdot \nabla T+\rho C_{p}\frac{\partial T}{\partial t}=\nabla \cdot \left(k\nabla T\right)+Q,$$

where *C*_p_ is the specific heat capacity, *T* is the temperature, *k* is the thermal conductivity, and **Q** is the heat source.

The growth of the cladding layer adopts the method of mesh deformation. The normal growth rate of the interface mesh is defined as the growth rate of the cladding interface. The governing equation can be described as:

(7) $$V_{p}=\frac{2m_{f}\eta_{m}}{\rho_{m}\pi r_{p}^{2}}\exp\left(\frac{-2\left({\left(x-v_{s}t\right)}^{2}+y^{2}\right)}{r_{p}^{2}}\right)z,$$

where *m*_f_ is the mass flow rate, *η*_m_ is the powder catchment efficiency, *r*_p_ is the mass flow radius, and **z** is the unit vector in the **z** direction.

### 3.3. Boundary Conditions and Calculating Parameters

The initial temperature on the bottom surface is set as room temperature. On the lateral direction of the specimen, heat loss is modeled by convection and radiation:

(8) $$k\nabla T=-h\left(T-T_{0}\right)-\varepsilon \sigma \left(T^{4}-T_{0}^{4}\right),$$

where *h* is the heat transfer coefficient, *T*_0_ is the ambient temperature, *ε* is the radiation coefficient, and *σ* is the Stefan–Boltzmann constant.

**Figure 2:**
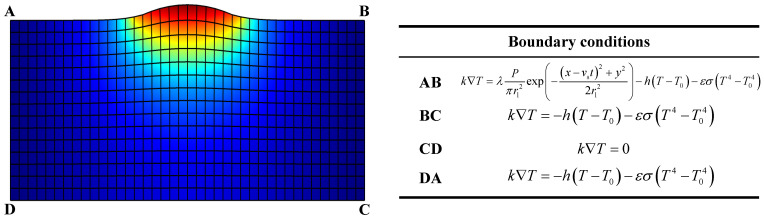
Boundary conditions in the numerical model.

In addition to the convection and radiation on the top surface, a laser heat source is involved in the model:

(9) $$k\nabla T=\lambda \frac{P}{\pi r_{l}^{2}}\exp\left(-\frac{{\left(x-v_{s}t\right)}^{2}+y^{2}}{2r_{l}^{2}}\right)-h\left(T-T_{0}\right)-\varepsilon \sigma \left(T^{4}-T_{0}^{4}\right),$$

where *λ* is the absorptivity of laser, *P* is the power of laser, and *r*_l_ is the radius of the spot vs. the scanning speed. The boundary conditions are shown in Figure 2.

Each simulation started when the laser began to act on the cross-section, and it stopped when the laser completely moved away. Regarding the area change of the heat source caused by the moving of the laser, the heat source in the 2D model was assumed as a time-varying line heat source with Gaussian energy distribution. The 3D characteristics of the heat source were considered by using the above assumptions, which were also used in the references [25,26,27,28].

Vibration was applied by pressure boundary in the numerical simulation. The 1D vibration was applied by setting a pressure boundary at the bottom of the substrate, corresponding to the experiments with vertical vibration. Furthermore, the 2D vibration was applied by setting pressure boundaries at the bottom and both sides of the substrate, corresponding to the experiments with vertical + horizontal vibration. The pressure generated by the vibration was expressed as:

(10) $$P_{v}=2\pi f\rho cA\cdot \cos\left(2\pi ft_{1}\right),$$

where *f* is the vibration frequency, *A* is the amplitude, *c* is the propagation speed of vibration in the specimen, and *t*_1_ is the vibration action time.

The material properties and the laser process parameters used in the numerical simulation are shown in Table 2 and Table 3, respectively.

## 4. Results

### 4.1. Flow Field and Temperature Field

**Figure 3:**
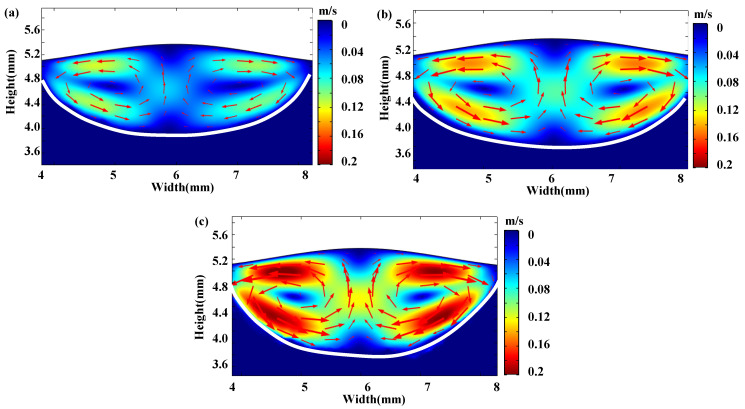
The flow field distribution of different vibration modes in the laser cladding melt pool at *t* = 0.45 s: (**a**) without vibration; (**b**) with 1D vibration; (**c**) with 2D vibration.

**Figure 4:**
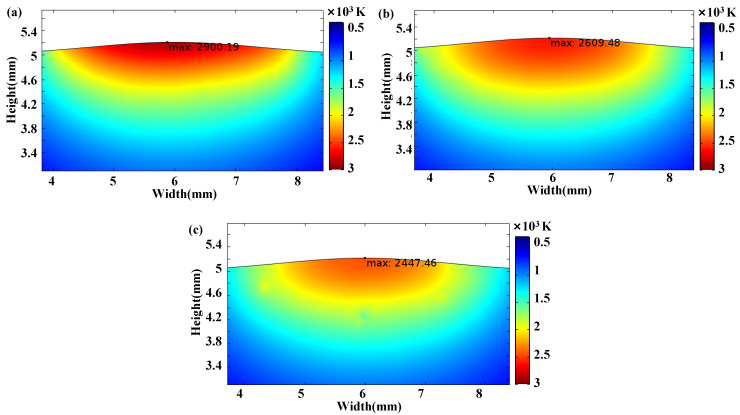
Temperature fields in the cooling process at the same time: (**a**) without vibration; (**b**) with 1D vibration; (**c**) with 2D vibration.

The flow fields and temperature fields in the molten pool of the cross-section with and without vibration at *t* = 0.45 s are shown in Figure 3 and Figure 4, respectively. It can be observed from Figure 3 that the velocity of the melt flow in the molten pool was promoted by the vibration, while the flow tendency of the circulation was not changed. The maximum flow velocity without vibration was 0.011 m/s in the molten pool. On the other hand, the flow velocity was 0.015 m/s, 0.021 m/s for 1D vibration and 2D vibration, increasing by 36% and 91%, respectively. It can be concluded that convective intensity in the molten pool was promoted remarkably due to the oscillation and stirring caused by the vibration.

The temperature of the substrate decreased when the laser beam moved away. The cooling processes were influenced by the vibration, and the results are shown in Figure 4. When the laser beam moved away for 0.1 s, the maximum temperatures were 2900.19 K, 2609.48 K and 2447.46 K at the conditions without vibration, with 1D vibration and 2D vibration, respectively. It indicated that the convective heat flux and the cooling rate were both effectively improved with the applying of vibration. Furthermore, the effect was more significant with the 2D vibration.

### 4.2. Microstructure Characteristics

**Figure 5:**
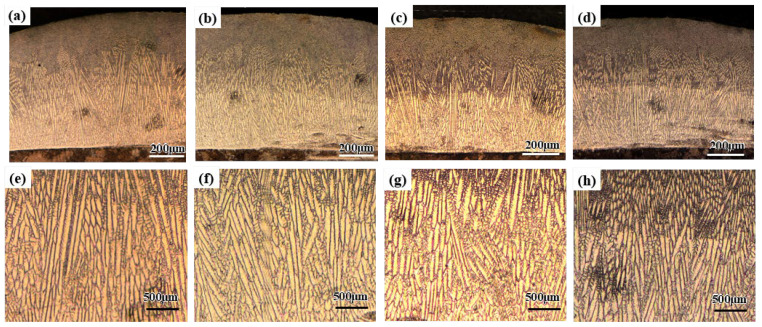
Cross-sectional morphology of laser cladding with or without vibration: (**a**) without vibration; (**b**) with 1D vibration; (**c**) with 2D vibration; (**d**) with 3D vibration; (**e**) without vibration in high magnifications; (**f**) with 1D vibration in high magnifications; (**g**) with 2D vibration in high magnifications; (**h**) with 3D vibration in high magnifications.

**Figure 6:**
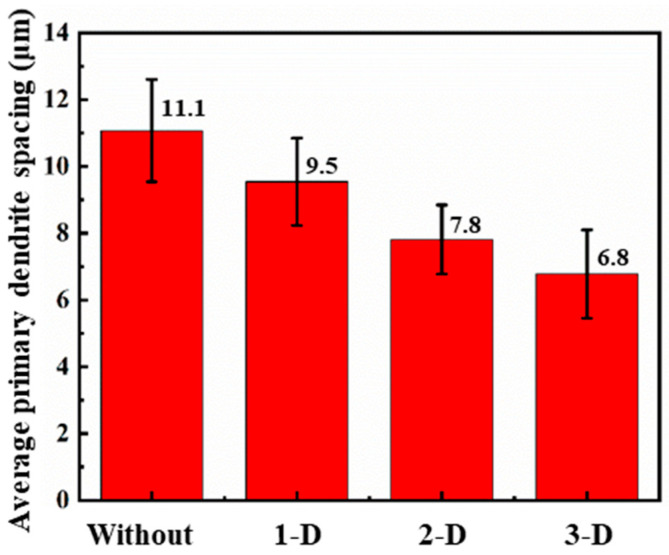
Comparison of average primary dendrite spacing in the middle of cladding layer with and without vibration.

During the laser cladding with and without vibration, the substrate and the powders were melted quickly into the molten pool with the radiation of laser beam. The solidification started from the bottom of the molten pool and then all the way to the top. The cross-sectional morphology of laser cladding with and without vibration is shown in Figure 5. It can be observed that the microstructures of the cladding layers were dense and uniform. In addition, there was no significant defects in the cladding layer and the bonding zone. Epitaxial columnar dendrites can be observed in the cladding layers with and without vibration. However, the size and growth direction of columnar dendrites varied with high-frequency vibration. In addition, the comparison of average primary dendrite spacing in the middle of the cladding layer with and without vibration is shown in Figure 6. The average primary dendrite spacings were 11.1 μm, 9.5 μm, 7.8 μm and 6.8 μm at the conditions without vibration, with 1D vibration, 2D vibration and 3D vibration, respectively.

### 4.3. Microhardness and Tensile Properties

**Figure 7:**
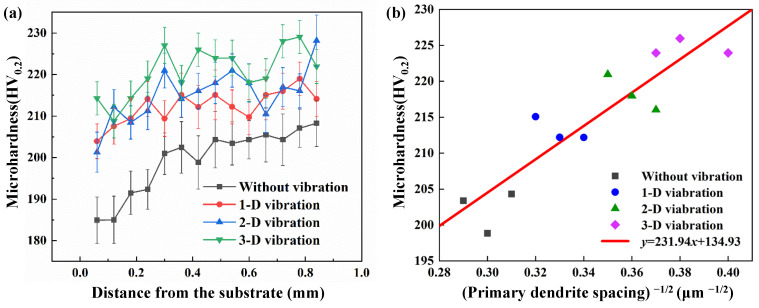
Microhardness distribution and the relationship between the microhardness and the primary dendrite spacing: (**a**) microhardness distribution along the build direction of the laser cladding layer with and without vibration; (**b**) the relationship between the microhardness and the primary dendrite spacing.

Vickers hardness was tested to investigate the effect of vibration on the mechanical properties of the cladding, and the results are shown in Figure 7a. The microhardness varied from 184 to 208 HV_0.2_ from the top to the bottom of the cladding without vibration, and the average microhardness was 199 HV_0.2_. When multidimensional high-frequency vibration was applied, the microhardness of the cladding layer was significantly improved. The average microhardness of the cladding layer was 212 HV_0.2_, 215 HV_0.2_, 221 HV_0.2_ for 1D, 2D, and 3D vibration, increasing by 6%, 8%, and 11%, respectively. It was commonly accepted that the microhardness increases significantly due to the refined microstructures [29,30]. Meanwhile, the microhardness fluctuation along the build direction was relatively minor with the assistance of vibration. In addition, the solid-solution strengthening can be promoted by the external vibration energy [15]. Therefore, the uniformity of the hardness increased with the applying of vibration. To further analyze the relationship between the microhardness and primary dendrite spacing, a Hall–Petch kind of relationship was well fitted, as shown in Figure 7b. It indicates that the decrease in the primary dendrite spacing caused by vibration is able to enhance the microhardness.

**Figure 8:**
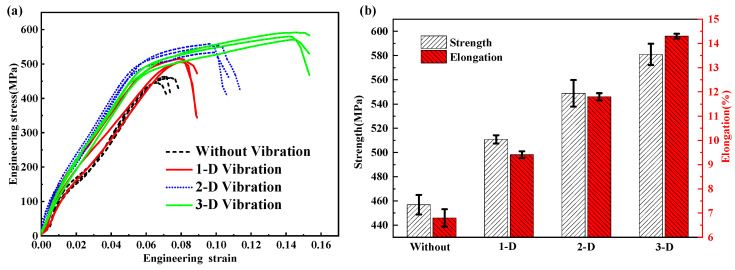
Tensile properties with various vibration conditions: (**a**) stress–strain curves of the axial tensile tests; (**b**) comparison of the tensile strength and elongation.

The tensile properties assisted by vibration are shown in Figure 8. It can be observed that the plasticity of samples with vibration was significantly enhanced. The stress–strain curves of the cladding layers are shown in Figure 8a. It can be found that the tensile strength was significantly improved by vibration. Furthermore, comparison of the tensile properties including tensile strength and elongations was carried out, and the results are shown in Figure 8b. The average tensile strength of the samples without vibration was 456.9 MPa, and the samples with 1D vibration, 2D vibration, and 3D vibration were 510.8 MPa, 548.8 MPa, and 580.9 MPa, increasing by 11.8%, 20.1%, and 27.2%, respectively. In addition, large elongations of samples were observed in the condition with vibration. The average elongations of the samples without vibration was only 6.80%, while the ones with 1D vibration, 2D vibration, and 3D vibration were 9.41%, 11.80% and 14.30%, increasing by 38.6%, 73.9%, and 110.8%, respectively.

**Figure 9:**
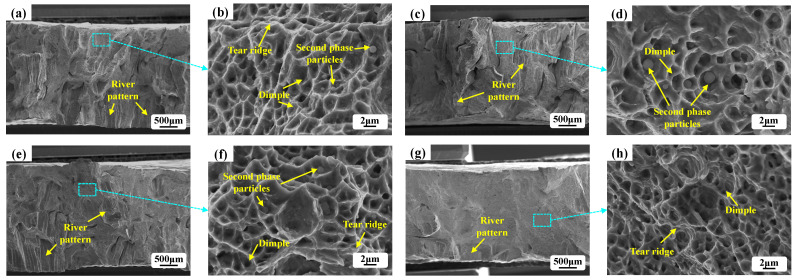
Fracture morphologies of the 316L samples after tensile tests: (**a**) without vibration in low magnifications; (**b**) without vibration in high magnifications; (**c**) with 1D vibration in low magnifications; (**d**) with 1D vibration in high magnifications; (**e**) with 2D vibration in low magnifications; (**f**) with 2D vibration in high magnifications; (**g**) with 3D vibration in low magnifications; (**h**) with 3D vibration in high magnifications.

The tensile fracture morphologies of the samples with various vibration conditions are shown in Figure 9. A combination of dimple and river pattern can be observed. It indicated that ductile fracture and cleavage fracture both occurred during the process of tensile fracture. Furthermore, it was found that the dimple was more significant while the river pattern showed an inverse result in the samples with 3D vibration. Namely, more ductile fracture and less cleavage fracture occurred in the samples with 3D vibration.

## 5. Discussion

In this study, a 2D numerical model was applied to simulate the cross-section of the laser cladding. Although there are limitations for the 2D simulation, useful information in the molten pool, including the temperature field and the flow field, etc., is able to be obtained from the 2D simulation with reasonable assumption and treatment [25]. It can be found that the increase of vibration dimension resulted in more significant oscillation and stirring in the molten pool, leading to the more significant effect on the flow field and temperature field with 2D vibration rather than 1D vibration, as shown in Figure 3 and Figure 4. Thus, it is reasonable to presume that the convective intensity in the molten pool is promoted more remarkably with 3D vibration. It was commonly accepted that the convective driving forces of the melt in the molten pool originated from the surface tension and the buoyancy force [31,32]. As shown in Figure 3, the convective tendency did not change because the direction of the surface tension and the buoyancy force did not vary with vibration. Furthermore, an additional oscillating force caused by the pressure difference was applied on the molten pool, leading to the increase in the convection intensity.

The solidification process of metal melt is directly affected by thermodynamics, kinetics and the cooling rate. The microstructure is affected by the solidification process. In this study, typical planar crystal structures columnar dendrites with different growth directions were observed in the cladding layer. It is commonly accepted that the primary dendritic spacing can be refined substantially with the thermal undercooling induced by the high cooling rate, while the dendrite structure is determined by the temperature gradient and solidification rate [33,34,35]. The heat dissipation direction also plays an important role in the forming of the dendrite structure [36]. The solidification temperature decreases with the increase in the cooling rate caused by the acceleration of the melt flow. According to the theory of metal solidification heat transfer, the relationship between primary dendritic spacing *D* and cooling rate *v* meets the law [37,38,39]:

(11) $$D=k\cdot v^{\beta },$$

where *k* and *β* are constants related to the material, and *k* is positive while *β* is negative.

It can be obtained from Equation (11) that primary dendritic spacing is inversely proportional to the cooling rate. Therefore, the larger cooling rate caused by vibration will lead to smaller primary dendritic spacing. With 1D high-frequency vibration, the cooling rate is affected by a single-dimensional oscillation applying to the molten pool. When multidimensional high-frequency vibration is applied, the melt flow can be accelerated and the heat transfers from the molten pool to the liquid–gas interface quickly. Significant stir occurs in 3D high-frequency vibration, leading to further accelerated cooling, dendrite fragmentation and irregular heat dissipation direction [19]. The above deduction is consistent with the experimental results in Figure 6 and the simulation results in Figure 3 and Figure 4.

The strength enhancement of the laser cladding depends on improvement of the microstructure [40]. The presence of even insignificant micropores may substantially reduce the coating’s strength [41,42]. The effect of vibration on porosity was investigated in the authors’ previous work [19]. In this study, however, the laser cladding process parameters were optimized, resulting in no obvious pores in the samples for tensile tests. With the assistance of multidimensional high-frequency vibration, the compactibility, strength and toughness of the samples are significantly improved due to a comprehensive effect by vibration, including the refinement of primary dendritic spacing, great metallurgical bonding of interface and the effect of solid-solution strengthening [15,43]. The larger cooling rate caused by vibration releases more latent heat of the melt during solidification [44]. Then, the primary dendritic spacing is refined and leads to the increase in microhardness shown in Figure 7b. An enhancement of wettability between the melt and substrate caused by oscillation and stirring of the molten pool leads to great compactibility and metallurgical bonding. In addition, the solid-solution strengthening of Fe is also promoted by the oscillation and stirring of the molten pool. Then, small and shallow dimples can be suppressed in the tensile tests suggesting the plasticity improvement of the cladding layer [45], which is consistent with the increase in the average elongation in Figure 8.

## 6. Conclusions

The microstructure and mechanical properties of multidimensional high-frequency vibration-assisted laser cladding were investigated in this study. Laser cladding with and without vibration was performed to uncover the benefits of multidimensional high-frequency vibration. A numerical model was established and analyzed. In the condition with vibration, the melt flow of molten pool was accelerated, and the temperature in the cooling process decreased more slowly. Moreover, the microstructure and microhardness were influenced significantly by multidimensional high-frequency vibration. The primary dendrite spacings decreased from 11.1 to 9.5 μm, 7.8 μm and 6.8 μm with 1D, 2D and 3D vibration, respectively. The microhardness of samples with 1D, 2D and 3D vibration increased by 6%, 8%, and 11%, respectively. Tensile properties including tensile strength and elongation were also improved significantly. The larger and deeper tearing dimples can be observed in the tensile fracture samples with vibration, which suggested plasticity improvement of the cladding layer. The major source of property improvement was explained by a combined effect of oscillation and stirring, and 3D vibration in this study proved to be a much more efficient method to improve the microstructure and mechanical properties than 1D, 2D and without vibration.

## Figures and Tables

**Table 1:** Chemical compositions of 1045 steel substrate and SS316L powder (wt %).

	C	Mn	Mo	Si	Cr	Ni	Fe
1045 steel	0.42–0.50	0.50–0.80	/	0.17–0.37	≤0.17	≤0.30	Bal.
SS316L	<0.03	≤2	2–3	≤1	16–18	12–15	Bal.

**Table 2:** Material properties used in the numerical simulation.

Parameter	Symbol	Value	Parameter	Symbol	Value
Density	*ρ*	7870 kg/m^3^	Dynamic viscosity	*μ*	0.04 Pa·s
Melting point	*T*_ref_	1856 K	Solidus temperature	*T*_s_	1846 K
Solid specific heat	*cp*_s_	460 J/(kg·K)	Liquidus temperature	*T*_l_	1866 K
Liquidus specific heat	*cp*_l_	594.7 J/(kg·K)	Emissivity	*ε*	0.6
Thermal conductivity	*k*	50 W/(m·K)			

**Table 3:** Laser process parameters used in the numerical simulation.

Parameter	Symbol	Value	Parameter	Symbol	Value
Initial temperature	*T*_0_	293.15 K	Laser beam radius	*r*_l_	4 mm
Laser power	*P*	1600 W	Mass powder rate	*m*_f_	6 g/min
Scanning speed	*v*_s_	5 mm/s	Vibration frequency	*f*	2196 Hz
Amplitude	*A*	10 μm			

## Data Availability

Not applicable.
